# Microfluidic Synthesis of Microfibers for Magnetic-Responsive Controlled Drug Release and Cell Culture

**DOI:** 10.1371/journal.pone.0033184

**Published:** 2012-03-28

**Authors:** Yung-Sheng Lin, Keng-Shiang Huang, Chih-Hui Yang, Chih-Yu Wang, Yuh-Shyong Yang, Hsiang-Chen Hsu, Yu-Ju Liao, Chia-Wen Tsai

**Affiliations:** 1 Department of Applied Cosmetology and Master Program of Cosmetic Science, Hungkuang University, Taichung, Taiwan; 2 School of Chinese Medicine for Post-Baccalaureate, I-Shou University, Kaohsiung, Taiwan; 3 Department of Biological Science and Technology, I-Shou University, Kaohsiung, Taiwan; 4 Department of Biomedical Engineering, I-Shou University, Kaohsiung, Taiwan; 5 Department of Biological Science and Technology, National Chiao Tung University, Hsinchu, Taiwan; 6 Department of Mechanical and Automation Engineering, I-Shou University, Kaohsiung, Taiwan; 7 Department of Information Management, Ming Chuan University, Taipei, Taiwan; University of Queensland, Australia

## Abstract

This study demonstrated the fabrication of alginate microfibers using a modular microfluidic system for magnetic-responsive controlled drug release and cell culture. A novel two-dimensional fluid-focusing technique with multi-inlets and junctions was used to spatiotemporally control the continuous laminar flow of alginate solutions. The diameter of the manufactured microfibers, which ranged from 211 µm to 364 µm, could be well controlled by changing the flow rate of the continuous phase. While the model drug, diclofenac, was encapsulated into microfibers, the drug release profile exhibited the characteristic of a proper and steady release. Furthermore, the diclofenac release kinetics from the magnetic iron oxide-loaded microfibers could be controlled externally, allowing for a rapid drug release by applying a magnetic force. In addition, the successful culture of *glioblastoma multiforme* cells in the microfibers demonstrated a good structural integrity and environment to grow cells that could be applied in drug screening for targeting cancer cells. The proposed microfluidic system has the advantages of ease of fabrication, simplicity, and a fast and low-cost process that is capable of generating functional microfibers with the potential for biomedical applications, such as drug controlled release and cell culture.

## Introduction

Currently, most drug delivery carriers are micro-/nanoparticles [1], [2], [3]. These spherical dosage forms have been studied extensively for their drug release profiles, but some limitations still exist. For example, they are easily expelled from the target site and that they have a high initial burst release rate [4], [5]. The structure of the microfibers is a potentially alternative dosage form for obtaining a controlled zero-order release profile [6]. By providing a continuous structural integrity, microfibers can be new carriers for delivering delicate compounds such as water soluble drugs that have low encapsulation efficiency and reduced bioactivity in conventional vehicles [7], [8], [9].

Melt spinning, wet spinning, and electrospinning are common methods to produce microfibers [10], [11], [12], [13]. However, melt spinning needs bulky and heavy equipment for its high temperature process, while wet spinning involves volatile organic solvents, rendering them unacceptable for protein encapsulation. The microfibers produced by electrospinning are difficult to align directionally, and too thin to have a high enough mechanical strength for three-dimensional (3D) scaffolds applications [14], [15]. In contrast, microfluidic technology is simple, cost-effective, is compatible with biological materials and thus an alternative method for producing uniform micro-/nanofibers [14], [16], [17], [18].

Numerous studies have utilized magnetic nanoparticles for medical and biological applications, such as drug/gene delivery, bio-separation, and magnetic resonance imaging [19], [20], [21], [22], [23]. Previous studies have demonstrated that magnetic nanoparticles can be controlled to facilitate drug release from spherical particles [24], [25], [26]. This suggests that magnetic nanoparticles can be entrapped in microfibers. However, this kind of magnetically-controlled release strategy has not yet been applied on microfibers.

Therefore, the aim of this study was to develop a facile way to obtain polymer microfibers by utilizing microfluidic technology. In order to have good control over the fabrication of the microfibers, we used the design of multi-inlets and multi-junctions to achieve the dispersed and the continuous phases for solidifying and shielding the microfibers without them clogging the microchannels [16]. The advantage of the microchannel design is that it is simple and efficient to fabricate microfibers using a one-step continuous process. The main novelties of this study are (i) a linear release behavior from the diclofenac-loaded microfibers; (ii) an active control over the drug release rate from the microfibers by exerting magnetic iron oxide (MIO) nanoparticles; and (iii) a one-step cell encapsulation system to culture cancer cells for screening potential anticancer agents.

## Materials and Methods

### 1. Materials

Sodium alginate (cat. A2158), diclofenac sodium (cat. D6899), calcium chloride (cat. 499609), and magnetic iron oxide (MIO) nanoparticles (cat. 725331) were purchased from Sigma-Aldrich Co. (St. Louis, MO). Sunflower seed oil was obtained from Uni-President Enterprise Co. (Taiwan). De-ionized water was prepared using a Milli-Q® system (Millipore Inc., Clifton, NJ). *Glioblastoma Multiforme* (GBM) cells used in the present study were obtained from the Bioresource Collection and Research Center (BCRC) in Taiwan. All other reagents were commercially available and of analytical grade.

### 2. Design and fabrication of a microfluidic chip

**Figure 1:**
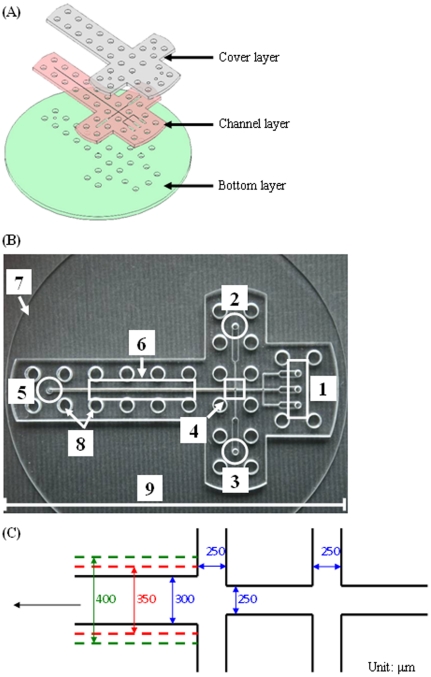
Microfluidic chip. Expanded view (A) and a photo (B) of the microfluidic chip: 1, inlets of center channels; 2 and 3, inlets of side channels; 4, cross junction; 5, outlet; 6, observation channel; 7, bottom layer disk; 8, screw orifices; 9, the scale bar = 11 cm. (C) is the geometry of the microfluidic channels.

The microfluidic chip was designed by AutoCAD® 2007 (Autodesk, USA). The CAD data was then fed to a computer-controlled CO_2_ laser machine (LaserPro Venus, GCC, Taiwan) for the laser micromachining process and laid out on a conventional poly methyl methacrylate (PMMA) substrate (length/width/depth: 270/210/1.5 mm) [27], [28], [29], [30], [31]. As shown in the expanded view of **Fig. 1A**, the microfluidic chip consists of three layers (length/width/depth: 110/45/4.5 mm) which included, from top to bottom: the cover layer (with reagent inlet), the channel layer (cross-junction microchannels) and the bottom layer (with product outlet), respectively. These three layers were held together by twenty-nine M4 screws, and were tightened at 1∼1.2 Nm using an adjustable torque wrench. The microfluidic chip has five inlets (three for the center channels and two for the side channels), two cross junctions and one outlet, as shown in **Fig. 1B**. The broadened channel near the outlet is the observation channel which was designed for slowing down the flow and enhancing the observation of the analysis (**Fig. 1C**). There are three types of downstream channels (300, 350 and 400 µm, respectively) in this study

### 3. Manufacturing procedure of microfibers

**Figure 2:**
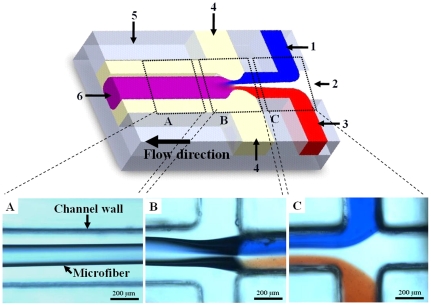
Microfiber formation. The diagram of the microfluidic system and photographs of observation positions. 1, 2 wt % CaCl_2_ solution; 2, deionized water; 3, alginate solution; 4, sunflower seed oil; 5, observation channel; 6, microfibers. The formation of microfibers: A, photograph of the microfiber in the observation channel; B, Photograph of the second cross junction; C, photograph of the first cross junction.

The dispersed phase was composed of 2 wt. % CaCl_2_, deionized water, and 1 wt. % alginate solutions. In order to enhance observation of the synthesized microfibers, we incorporated 0.1 wt. % Rhodamine B in the alginate solution in some of the experiments. As shown in **Fig. 2**, the alginate and CaCl_2_ solutions were injected into two of the central side channels, respectively, and sandwiched the deionized water thereby retarding the gelling of the alginate at the first junction. The sunflower seed oil as the continuous phase was delivered from two side channels at the second junction. All solutions were connected to the microfluidic platform by Teflon tubing with an I.D. of 0.76 mm, an O.D. of 1.22 mm, and a length of 400 mm. Simultaneous injection of the dispersed and continuous phases into the microfluidic chip was carried out by individual digitally controlled syringe pumps (KDS230, KD Scientific, USA). By tuning the flow rates of the dispersed and continuous phases, the microfiber could be synthesized. The microfibers fabricated in the collected reservoir beneath the outlet of the microfluidic chip were retrieved for further examination (**Text S1**). The stability of the microfibers under different pH conditions was described in the **Text S2** (please see **Fig. S1** about microscopic images of the microfibers).

### 4. Release behavior of the diclofenac-loaded microfibers with/without MIO nanoparticles

Diclofenac was used as the model drug to estimate the drug release behavior of the microfiber. To enhance and to control the release efficiency of the microfibers, MIO nanoparticles with an average 5 nm particle size were loaded into the microfiber. The alginate solution consisted of 1 wt. % alginate, 1 wt. % diclofenac sodium, and 4 wt. % MIO nanoparticles. The encapsulation efficiency (EF) of the MIO nanoparticles detected by inductively coupled plasma mass spectrometry was determined indirectly according to the literature [32]. The amount of MIO nanoparticles entrapped in the microfibers was calculated by the difference between the total amount used to prepare the systems and the amount of MIO nanoparticles that remained in the aqueous phase after isolating the microfibers.
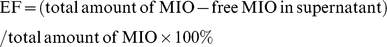



In the release study, 0.1 g manufactured microfibers containing diclofenac and MIO nanoparticles were dispersed in 1 mL pH 7.4 and 37°C water solutions. The proposed release system was conducted on an orbital shaker at a constant shaking of 100 rpm. An external magnetic field, 0.4 Tesla (T) at the border of the magnet, was applied on the sample by a 0.5 cm distance at determined times as the release experiment was carried out. At pre-determined time intervals, 10 µL solutions were drawn to determine the concentration of diclofenac by high-performance liquid chromatography (HPLC) analysis (L2000 system, Hitachi, Japan).

### 5. Cell culture in the microfiber

The characteristics of biocompatibility, biodegradability, high water content, and tissue-like elasticity make alginate an ideal candidate for scaffolds when growing cells and tissues. Similar to the process of preparing diclofenac-loaded microfibers, 1.65×10^5^ cells/mL GBM cells were used as model cells to be encapsulated in microfibers for studying the cell culture in vitro. The GBM cell-contained microfibers were cultured in Dulbecco's modified media (DMEM) supplemented with 10% (v/v) fetal bovine serum (FBS) at 37°C with 5% CO_2_ in a humidified incubator. At pre-determined times, the GBM cells in the microfibers were observed through the microscope.

## Results and Discussion

### 1. Principle of the formation of microfibers

Based on the external crosslinking process of alginate [33], we created a microfluidic system to control the spontaneous self-assembly of alginate microfibers. As shown in **Fig. 2**, the components of the dispersed phase, containing a CaCl_2_ gelating solution, deionized water, and a sodium alginate solution, were injected into the microchannels individually. Sodium alginate was crosslinked and then solidified to calcium alginate by diffusion of the Ca^2+^ ions to exchange sodium ions from the guluronic acids with the divalent cations [33]. The design of three-in dispersed phase has the advantage that it makes it easy to control the three individual injections to solidify alginate microfibers by gradual extrusion without clogging. After the components of the dispersed phase were mixed, the microfiber formed. The continuous oil phase then shielded the microfiber after the second junction to prevent aggregation on the channel wall.

### 2. Synthesis of microfibers

**Figure 3:**
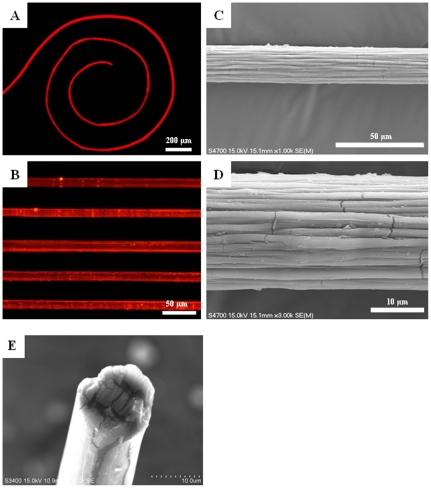
Microfiber images. Microscopic images (A∼B, stained with Rhodamine B) and scanning electron microscopy images (C∼E) of microfibers.

Alginate, commonly extracted from brown *algae*, is a natural biopolymer and advantageous by being non-immunogenic, biocompatible, and able to dissolve and degrade under physiological conditions [34], [35]. It is one of the most employed and recognized material for the applications of drug delivery and cell encapsulation [36], [37], [38]. Alginate can be cross-linked by divalent cations to form gel structures as requested, and this tailor-made property makes it advantageous as a formulation aid. A dispersed phase consisting of CaCl_2_, deionized water and alginate solutions was delivered into the three center channels to generate alginate microfibers. As shown in **Fig. 2**, the CaCl_2_ (stained with methylene blue) and alginate solutions (stained with Rhodamine B - red color) were separated by water, which allowed the formation of microfibers to occur without immediate gelation. It was found that the three solutions of the dispersed phase resulted in a laminar flow after the first cross junction (**Fig. 2C**) which was then compressed by the sheath force of the oil at the second cross junction (**Fig. 2B**); meanwhile the Na^+^ ions from the sodium alginate were exchanged by Ca^2+^ ions, resulting in the formation of cross-linked alginate microfibers (**Fig. 2A**). The microfibers formed continuously without breaking off in the chip microchannels (**Fig. 3A**), and further gelation was not needed. The resulting microfibers were cylindrical with a uniform diameter (**Fig. 3B**). Scanning electron microscope (SEM) analysis of microfibers revealed that the surface of the microfibers appeared to have bundle-cluster morphology (**Fig. 3C–D**).

This microfluidic platform successfully manufactured alginate microfibers for the applications mentioned below. The proposed microfluidic chip features a design of multi-inlets and multi-junctions that deliver immiscible phases to solidify and shield the alginate microfibers continuously and smoothly without breaking off or clogging.

### 3. Influence of flow rates and observation channel width on fiber size

To further understand the process we studied the relationships among the microfiber diameter, the flow speed of the continuous phase (sunflower oil) and the width of the observation channel. As shown in **Table 1**, the diameter of the microfibers decreased from 364.3±16.3 to 205.3±5.7 µm when the flow speed of the continuous phase increased from 1 to 5 mL/min with an observation channel width of 0.3 mm. The same phenomenon was noted at observation channels with widths of 0.35 and 0.4 mm, respectively, indicating that the higher flow speed of the continuous phase results in significantly narrower microfibers (ANOVA test, p<0.01). Exploring the effects of the observation channel on the microfiber size revealed that there were no significant differences (ANOVA test, p>0.05) among the diameters of the microfibers obtained from different channel widths (0.3∼0.4 mm) at the same flow speed of the continuous phase.

**Table 1:** The relationships among the diameter of microfibers, width of observation channel and flow rate of continuous phase.

Observation channelwidth(mm)	Continuous phase(mL/min)	Microfiber diameter (µm)	
		Average size	RSD
0.3	1	364.3	16.3
	2	287.1	8.1
	3	245.4	9.2
	4	226.9	12.3
	5	205.3	5.7
0.35	1	341.0	5.7
	2	276.3	14.1
	3	230.0	10.7
	4	208.3	8.0
	5	177.4	2.5
0.4	1	345.7	9.6
	2	273.1	8.0
	3	242.3	7.1
	4	228.4	7.1
	5	211.4	7.1

The above mentioned results show that the flow speed of the continuous phase was an effective factor for the diameter of the microfibers, and can be used as a control parameter. However, the effect of the channel width was less pronounced than that of the flow speed of the continuous phase. Equations (1), (2), and (3) represent the relationships between the diameter of the microfibers and the flow speed of the continuous phase with the channel width of 0.3, 0.35 and 0.4 mm, respectively.
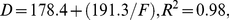
(1)

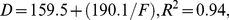
(2)

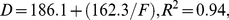
(3)where *D* = the diameter (µm), and *F* = the flow rate (mL/hour).

In addition, we studied the effects of the dispersed phase flow rate on the size of the microfibers. As shown in **Table 2**, the microfiber diameter increases with the dispersed phase flow rate, agreeing with previous literatures [16], [39]. The small relative standard deviation reflects the good uniformity of alginate microfibers obtained under each flow condition.

**Table 2:** The relationships between the diameter of the microfiber and the flow rate of the dispersed phase.

Observationchannel width (mm)	Continuous phase(mL/min)	Dispersed phase (mL/min)			Microfiber diameter (µm)	
		alginate	CaCl_2_	water	Average size	RSD
0.35	1	0.02	0.24	0.24	285.1	4.4
		0.04	0.45	0.45	323.9	5.8
		0.08	0.90	0.90	387.5	5.6

### 4. In vitro release of diclofenac

**Figure 4:**
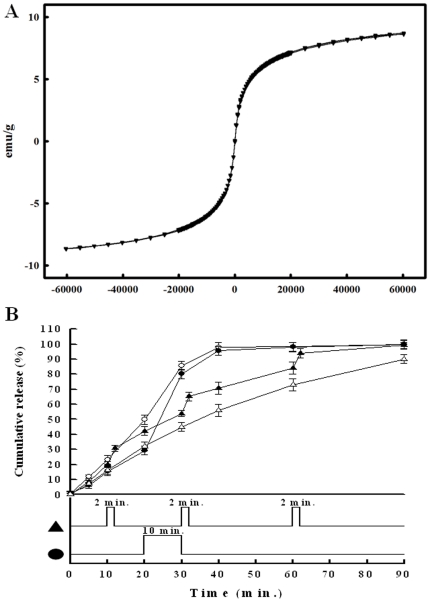
Characteristics of the microfibers. (A) The hysteresis curve of the microfibers containing MIO nanoparticles. (B) Release profiles of diclofenac from MIO-loaded microfibers without magnetic stimulation as the control (▵), with 2 minutes stimulation at the 10th, 30th and 60th minute (▴), with a 10-minute stimulation after the 20th minutes (•) and with a continuous stimulation from the beginning (○).

Diclofenac is a widely used non-steroidal anti-inflammatory drugs (NSAIDs) for local and systemic administration. A stable drug delivery rate can reduce dose-related adverse effects caused by the NSAIDs, such as gastrointestinal complications [40], [41]. To monitor the drug release behavior of the alginate microfibers, diclofenac was encapsulated in the microfibers for the release study. To study the drug release in relation to magnetic control, we first investigated the magnetism of the as-prepared microfibers containing magnetic nanoparticles. **Fig. 4A** shows the magnetization curve of the as-prepared microfibers as a function of the applied fields at 300 K. The saturation magnetization value of the microfibers is above 8 emu/g microfiber. As shown in **Fig. 4B**, the release of diclofenac from the microfibers without magnetic force was used as the control group. The release profile was approximately a linear release, and up to 91.9±2.8% of the encapsulated diclofenac was emptied after 1.5 hours. This indicated that the microfibers exhibited an excellent advantage as drug delivery carriers over the common microparticles which suffer from excessive initial burst release [4], [5]. The approximate zero-order release pattern of the microfibers offered a steady sustained release for pharmaceutical applications.

Except for leaking out of the microfibers by passive diffusion, the suitable active control approach of drug release can broaden the microfibers applications. The *in vitro* release data in triplicate shown in **Fig. 4B** reveals that the drug release profile of the resulting microfibers is controllable by a specific magnetic field. When applying a magnetic force from the beginning or at the 20^th^ minute for 10 minutes, 98.1±2.9% and 96.5±3.0% diclofenac released from microfibers at the 40^th^ minute, respectively. These two fast release rates are due to the fact that the MIO nanoparticles in the microfibers were attracted by the magnetic force and made the microfibers more porous. When applying the same magnetic force at the 10^th^, 30^th^ and 60^th^ minute for 2 minutes, burst releases were found when the magnetic force was applied. This result show that the microfibers encapsulating MIO nanoparticles were well made and that the release of drugs from the microfibers was enhanced and could be controlled externally. Therefore, fabricated microfibers can offer an advantage over conventional drug delivery devices by providing an improved dosing precision, ease of operation, and better compliance.

The encapsulation efficiencies of both diclofenac and the MIO nanoparticles were greater than 90%. The drug loading efficiency of external gelation depends on the material properties of the matrix substrate. The encapsulation efficiency of this proposed microfluidic method is similar to that of the traditional external gelation method [42]. Generally speaking, when the same material (alginate) is employed, a similar encapsulation efficiency is obtained.

### 5. Encapsulation of GBM cells

**Figure 5:**
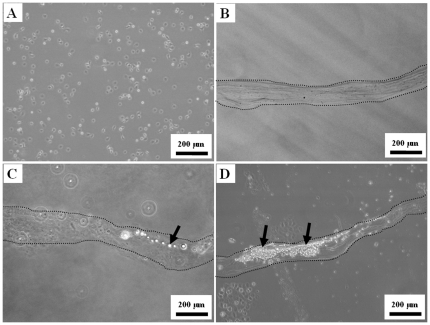
Cell culture of microfibers. Proliferation of GBM cells in microfibers. A. GBM cells; B. microfiber without cells; C. GBM in microfibers at the 1st day; D. GBM in microfibers at the 7th day. Arrows indicate GBM cells.

In addition to molecular drugs, microfibers can also encapsulate tumor cells for drug screening applications. The cell culture in the hollow fiber assay (HFA) developed by the US National Cancer Institute was proven to be a hastening method for screening potential anticancer agents [43], [44]. Our microfluidic system provided a one-step cell filling platform that saves a lot of human labor compared to the traditional HFA method, including tediously repeating flushing processes of solutions and cells [43], [44]. In the present study, GBM, which is the most common and lethal type of brain cancer [45], was chosen to demonstrate cell culture in the microfibers for drug screening to target cancer cells [46]. The GBM-loaded microfibers were cultured directly after the microfiber fabrication process. Because the microfluidic encapsulation process was mild and cell friendly, GBM cells (**Fig. 5A**) seemed not to be affected greatly by this manufacturing process and could adhere well to the microfibers (**Fig. 5B**) to undergo proliferation (**Fig. 5C & 5D**). It was evident that the GBM cells were arranged in tube-like structures and that direct cell to cell contact took place. The cell proliferation in the microfibers went with the culture time (**Fig. 5C & 5D**). The results demonstrated that the microfibers can provide a good environment for culturing cells. In addition to loading tumor cells for drug screening applications, these microfibers can also encapsulate cells for numerous tissue engineering or therapeutic applications such as nerve conduits, protection from immune rejection (immunoisolation) and for a bioreactor [47], [48], [49].

This study successfully proposed a simple microfluidic system for fabricating sophisticated microfibers. The diameter of these microfibers, ranging from 177.4 µm to 364.3 µm, could be easily controlled by regulating the flow rate and channel size. The diclofenac-loaded microfibers appeared to have a zero-order drug release, and this release rate could be actively controlled by applying magnetic iron oxide nanoparticles. The successful culture of *Glioblastoma Multiforme* cells verified that microfibers from this microfluidic system could provide a good environment for cell culture and tissue engineering applications.

## Supporting Information

Text S1 **Morphology and size measurement.**
(DOC)

Text S2 **Stability of the microfibers under different pH conditions.**
(DOC)

Figure S1 **Microscopic images of microfibers in different solutions.**
(DOC)
