# Supplementary material for: Microfluidic Synthesis of Microfibers for Magnetic-Responsive Controlled Drug Release and Cell Culture
Source: PLoS One. 2012 Mar 28;7(3):e33184. doi: 10.1371/journal.pone.0033184 (PMC3314645; doi:10.1371/journal.pone.0033184)
Supplement: Text S1 — Morphology and size measurement. (DOC) [file pone.0033184.s001.doc]

**Text S1. Morphology and size measurement**

An optical microscope system (TE2000U, Nikon, USA) and a digital camera (Evolution color VF, Nikon, USA) were utilized to observe the collected microfibers morphology. The recorded pictures were further analyzed by a personal computer with the homemade images analysis software in MATLAB (MathWorks Inc., USA) to determine the average diameter of the microfibers. Scanning electron microscopy (SEM, Hitachi S-2700, Japan) was used to evaluate the surface morphology of the microfibers. All data were expressed as the mean ± standard deviation, and statistical analysis between studied groups was performed using ANOVA test.
