# Supplementary material for: Microfluidic Synthesis of Microfibers for Magnetic-Responsive Controlled Drug Release and Cell Culture
Source: PLoS One. 2012 Mar 28;7(3):e33184. doi: 10.1371/journal.pone.0033184 (PMC3314645; doi:10.1371/journal.pone.0033184)
Supplement: Text S2 — Stability of the microfibers under different pH conditions. (DOC) [file pone.0033184.s002.doc]

**Text S2. Stability of the microfibers under different pH conditions**

**Fig. S1** investigates the effect of pH value on the stability of microfibers. The four tested solutions were double-distilled (DD) water (pH 7.2), gastric juice (pH 1.32), PBS (pH 7.44), and intestinal juice (pH 7.7). As shown in the microscopic images, the alginate microfibers remained almost unchanged in morphology for 9 hours in DD water and gastric juice. On the other hand, the alginate microfibers dissolved gradually in the PBS solution, and disintegrated after 9 minutes. It is evident that the disintegration of the microfibers occurred in a very short time in the intestinal juice. This result may be due to the electrostatic repulsion of carboxylic acids of alginate in an alkaline solution.
