# Supplementary material for: Microfluidic Synthesis of Microfibers for Magnetic-Responsive Controlled Drug Release and Cell Culture
Source: PLoS One. 2012 Mar 28;7(3):e33184. doi: 10.1371/journal.pone.0033184 (PMC3314645; doi:10.1371/journal.pone.0033184)
Supplement: Figure S1 — Microscopic images of microfibers in different solutions. (DOC) [file pone.0033184.s003.doc]

**
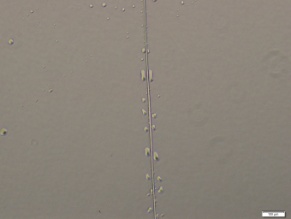

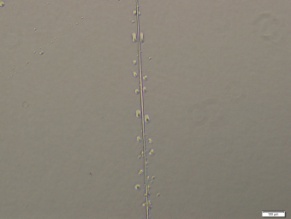

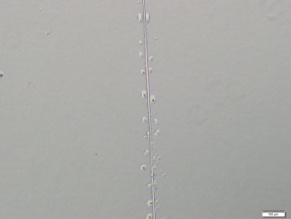
**

A

B

C

**
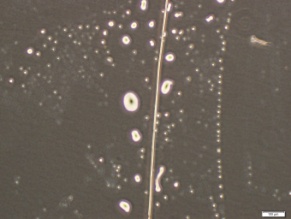

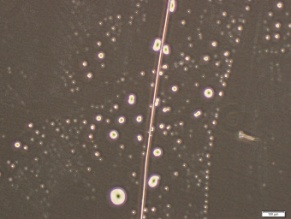

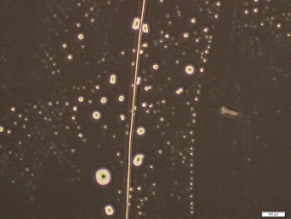
**

D

E

F

**
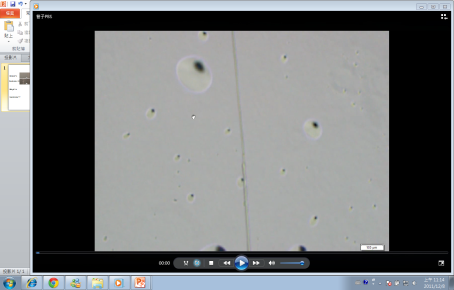

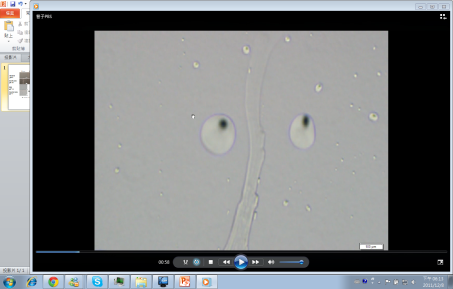

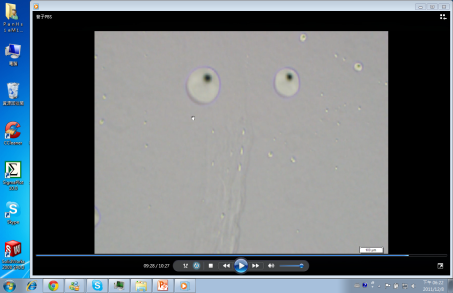
**

G

H

I

**
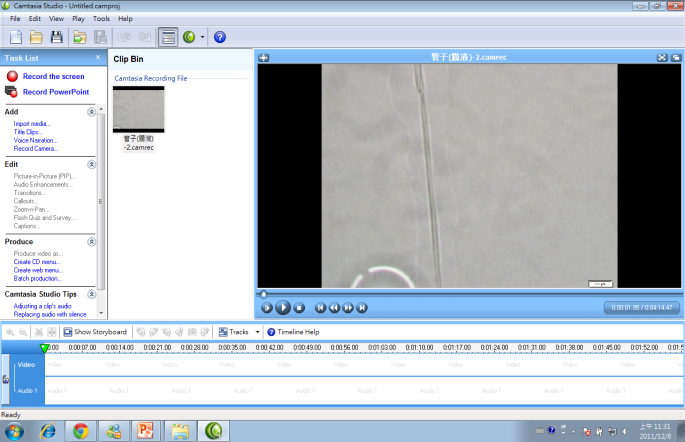

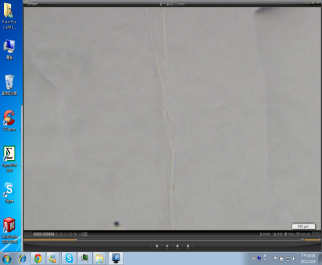
**

J

K

**Fig. S1**. Microscopic images of microfibers in different solutions. A~C are microfibers in DD water for 0, 3, 9 hours; D~F in gastric juice for 0, 3, 9 hours; G~I in PBS for 0, 0.5, 9 minutes; J~K in intestinal juice for 0 and 0.5 minutes. The scale bar is 100 μm.
